# HIV and Sexually Transmissible Infections among Money Boys in China: A Data Synthesis and Meta-Analysis

**DOI:** 10.1371/journal.pone.0048025

**Published:** 2012-11-29

**Authors:** Eric P. F. Chow, Ka I. Iu, Xiaoxing Fu, David P. Wilson, Lei Zhang

**Affiliations:** 1 The Kirby Institute, University of New South Wales, Sydney, Australia; 2 School of Sociology and Population Studies, Renmin University of China, Beijing, China; University of Southern California Keck School of Medicine, United States of America

## Abstract

**Background:**

Commercial sex workers within the population of men who have sex with men (MSM) in China, known as ‘money boys’ (MBs), are perceived to be at higher risk for HIV and other sexually-transmissible infections (STIs).

**Methods:**

We conducted a systematic review and meta-analyses from peer-reviewed literature accessed in two English (PubMed and Embase) and three Chinese databases (CNKI, CQVIP, Wanfang data). A data synthesis exercise was carried out to determine the extent and patterns of behaviours and HIV/STI epidemics. Pooled estimates, with 95% confidence intervals, for each study variable were calculated.

**Results:**

Thirty-two eligible articles (9 in English and 23 in Chinese) were identified. Our analysis indicated that Chinese MBs are generally young, currently employed, at low literacy levels and highly mobile. The prevalence of HIV, syphilis and co-infection among MBs were estimated to be 6.0% (4.2–8.5%), 12.4% (9.9–15.3%) and 2.2% (1.1–4.1%) over the period of 2004–2011. Level of condom use among MBs is generally higher than the broader MSM population (69.2% at last act, and 48.5% consistently over the past 6 months). One-third of the Chinese MBs identified themselves as bisexual and 8.7% (5.6–13.5%) are currently married to a female. Further, 40.9% (34.5–47.7%) of MBs participated in group sex in the past 12 months and 14.8% (10.6–20.3%) concurrently use illicit drugs.

**Conclusions:**

HIV/STI epidemics have affected Chinese MBs but the evidence suggests that the extent of infections is not greater than among other MSM in China.

## Introduction

The profile of HIV epidemics in China has been shifting. A distinct HIV epidemic has emerged among men who have sex with men (MSM) in China, a population of 10–20 million people [1], among whom HIV prevalence has been rapidly increasing from 1.5% in 2001 to 5.3% in 2009 [2]. MSM is an important population group in their own right, but there is also concern regarding their potential to act as a bridging population to their female sexual partners [3]–[5]. It is highly common for Chinese MSM in to marry for various reasons [6]. To reduce the spread of HIV among, and from MSM, it is valuable to understand the patterns and extent of behaviours and prevalence among MSM subpopulations.

‘Money Boys’ (MBs) are a subgroup of MSM who commercially sell sex to men. There are an estimated 380,000 MBs in China [7], many of whom are rural-to-urban migrants away from their hometown. Similar to the Chinese female sex workers (FSWs), MBs often enter the commercial sex industry for economic reasons [7] and consider their job of selling sex as temporary [7], [8]. However, due to their multiple male and female sexual partners and also unprotected sex [3]–[5], [8], MBs are potentially a core group for exacerbating the spread of HIV and other sexually transmitted infections (STIs) among MSM and for acting as a bridge to the broader heterosexual populations [1], [9]. Studies of MBs are limited in China and they are generally not included in routine HIV/STI surveillance. HIV/STI epidemiological and behavioural information on MBs has remained largely scattered among individual studies. There have been speculations that MBs have higher HIV/STIs prevalence than other MSM but there have been no studies to collate evidence on the risk behaviours and HIV/STI prevalence among MBs. This study aims to explore HIV/STI disease burden and associated sexual behaviours among Chinese MBs through a systematic review and meta-analysis.

## Methods

### Search strategy

A systematic review of studies published in English or Chinese was conducted through searches of the following electronic databases: PubMed, Embase, China National Knowledge Infrastructure (CNKI), Chinese Scientific Journals Fulltext Database (CQVIP) and Wanfang Data from 2000 to July 2012. The search was conducted using free-text terms and Medical Subject Headings (MeSH) terms: (‘money boys’ *OR* ‘MB’ *OR* ‘male sex workers’ *OR* ‘male commercial sex workers’) *AND* (‘human immunodeficiency virus’ *OR* ‘HIV’ *OR* ‘Acquired immune deficiency syndrome’ *OR* ‘AIDS’ *OR* ‘STD’ *OR* ‘STI’ *OR* ‘sexually transmitted disease’ OR ‘sexually transmitted infection’) *OR* (‘sexual behaviours’ *OR* ‘sexual partners’ *OR* ‘condom’ *OR* ‘unprotected sex’ *OR* ‘KAP’ *OR* ‘knowledge, attitude and practice’) *AND* (‘China’). This review was reported according to the PRISMA (Preferred Reporting Items for Systematic Reviews and Meta-Analyses) Statement issued in 2009 (Table S1) [10].

### Inclusion and exclusion criteria

Studies were eligible for inclusion in the review if they met the following criteria: (1) study published in Chinese or English language; (2) study subjects were Chinese MBs; (3) the objective of the study was investigating the HIV/STI epidemiology or risk behaviours among Chinese MBs; (4) HIV and STI diagnosis must be done in a laboratory with standard serologic testing methods. Studies were excluded if they met the following criteria: local/government reports and conference abstracts; and not original study, such as review papers. If the same study data were published in both English and Chinese sources, the articles published in Chinese language were excluded from the review. Two reviewers (EPFC, XF) independently screened the title, abstract and full-text. Disagreements of inclusion or exclusion criteria of the study were settled by a third investigator (LZ).

### Outcome of interest

In order to compare the demographic and behavioural characteristics between MBs and other MSM in China, identical indicators for Chinese MSM were collected from the latest available systematic review or meta-analysis at a national level. For indicators where such publications are absent, the latest cross-sectional study with the large sampling size (*n*>2,000) across multiple geographical regions in China would be used for background reference.

### Validity assessment

Eight items were used to assess the quality of studies: (1) clear definition of MBs; (2) representativeness of probability sampling; (3) sample characteristics matching the target population; (4) adequate response rate; (5) standardized data collection methods; (6) reliable survey measures; (7) valid survey measures; (8) appropriate statistical methods. All studies were rated on each indicator (1 for ‘Yes’ and 0 for ‘No’) for a total quality score between 0 and 8 (Table S2).

### Data extraction

Data from eligible studies were extracted into a database (Access, Microsoft, 2007). Data were extracted in four aspects: (1) study designs such as study year, sampling and recruitment methods; (2) demographic characteristics (Table 1); (3) HIV-related risk behaviours such as number of sexual partners, condom use with different types of sexual partnerships and percentage of MBs who participated in group sex (Table 2); and (4) HIV/STI epidemiology including prevalence of HIV and STI, laboratory testing methods and HIV testing rate (Table 3).

**Table 1:** Studies which reported the demographic and study characteristics among money boys in China.

First author, published year	Study Period	Study location (province)	Sample size (N)	Recruitment methodsφ	Sampling methods†	Years of selling sex	Employment status	Literacy level	Age range (mean±SD)	Percentage of migrants (%)	Participated in group sex activity (%)	Ever used drug (%)	Current married (%)	Self-identified sexual orientation*	Preferable sexual positioning‡	Quality assessment score
Cai YM, 2008 [37]	08/2004	Shenzhen (Guangdong)	138	Community	RDS	-	-	Junior or below = 42.0%Senior = 50.0%College or above = 5.8%	19–42 (23.5±4.1)	-	-	-	43.5%	B = 39.1%H = 4.4%G = 50.7%O = 5.8%	-	4
Cao NX, 2009 [38]	03/2006–06/2006	Chengdu (Sichuan); Shenyang (Liaoning); Nanjing (Jiangsu)	484	Not reported	Not reported	-	-	Junior or below = 44.8%Senior = 44.4%College or above = 10.7%	16–45 (21.6±3.0)	-	-	-	3.7%	-	-	3
Chen B, 2007 [39]	06/2007–10/2007	Chengdu (Sichuan)	42	Community outreach; MSM venues	Purpose	-	-	Junior or below = 40.5%Senior = 42.9%College or above = 16.7%	-	-	-	-	-	-	-	3
Chen B, 2011 [40]	05/2006–10/2008	Chengdu (Sichuan)	42	Not reported	Not reported	-	-	-	17–30	-	-	-	-	-	-	3
Cheng WB, 2010 [21]	05/2009–08/2009	Guangzhou (Guangdong)	151	Peer-referral, MSM venues	Purpose	≤1 yr = 60.9%;>1 yr = 39.1%		Junior or below = 41.7%Senior = 47.7%College or above = 10.6%	17–54	83.4%	-	13.9%	23.1%	B = 37.7%H = 3.3%G = 51.7%O = 7.3%	I = 21.8%R = 34.4%Both = 41.1%	6
Deng YH, 2009 [41]	11/2008–12/2008	Kunming (Yunnan)	45	Peer referral	Not reported	-	-	-	20–45 (26)	-	-	-	-	G = 100%	-	3
He N, 2007 [7]	2006	Shanghai	239	Community outreach; MSM venues	RDS; snowball	-	-	Junior or below = 41.8%Senior = 50.6%College or above = 7.1%	-	-	-	-	-	-	-	4
He N, 2007 [8]	2005	Shanghai	15	Not reported	Not reported	-	-	Junior or below = 13.3%Senior = 73.3%College or above = 13.3%	18–34	-	-	-	0.0%	-	-	3
Kong TSK, 2008 [3]	2004–2005	Beijing; Shanghai	30	Peer-referral	Snowball	6 mth–1 yr = 33.3%, 1–2 yr = 33.3%, 2–5 yr = 23.3%, >5 yr = 10%	Currently employed = 98.4%	Junior or below = 36.7%Senior = 43.3%College or above = 20.0%	18–32 (23.3)	-	-	-	3.3%	H = 10.0%G = 66.7%O = 23.3%	-	4
Lau JTF,2009 [20]	12/2007–02/2008	Shenzhen (Guangdong)	199	Community outreach	Not reported	<1 yr = 9.1%; 1 yr = 41.2%; 2 yr = 34.7%; > = 3 yr = 15.1%	-	Junior or below = 23.1%Senior = 52.3%College or above = 24.6%	N/A (22.41±2.25)	-	-	-	-	-	-	3
Li QH, 2006 [42]	01/2005–12/2005	Beijing	85	Clinics	Convenience	-	Currently employed = 99.4%	Junior or below = 24.7%Senior = 56.5%College or above = 18.8%	17–30 (22.25)	-	-	-	7.6%	G = 35.6%		4
Li Y, 2010 [43]	2004	Multiple locations (9 cities)	264	Peer referral	Snowball	-	-	-	-	-	-	-	-	B = 40.5%G = 44.7%	-	4
Li YF, 2012 [44]	03/2011–10/2011	Jining (Shandong)	41	Sentinel sites, VCT clinics and peer-referral	Not reported	-	-	-	N/A (22.23±3.3)	-	-	-	9.8%	G = 41.46%	-	5
Liu HJ, 2008 [6]	11/2007–12/2007	Shenzhen (Guangdong)	58	Peer-referral, MSM venues	RDS	-	-	Junior or below = 36.2%	18–44	-	-	21.0%	7.0%	-	-	4
Liu S, 2011 [1]	06/2009–10/2009	Shenzhen (Guangdong)	418	MSM venues	Time-location cluster sampling	0.9 years	-	Junior or below = 27.8%Senior = 52.2%College or above = 17.0%	N/A (23.6)	-	-	-	5.30%	B = 33.5%H = 25.4%G = 32.1%O = 9.1%	-	7
Liu ZQ, 2009 [45]	03/2007–06/2007	Tianjin	89	MSM venues	Not reported	-	-	Junior or below = 48.3%	16–29 (20.6)	93.3%	-	-	-	B = 51.7%H = 18%G = 29.2%O = 1.1%	-	4
Meng XD, 2010 [9]	10/2008–11/2008	Changchun (Jilin)	86	Not reported	Convenience	-	-	Junior or below = 38.4%Senior = 45.3%College or above = 16.3%	17–38 (24.6±3.22)	20.9%	-	-	24.4%	B = 20.9%G = 79.1%	-	3
Qi SZ, 2006 [46]	Not reported	3 cities in 2 provinces	95	MSM venues	RDS	1–72 months (mean = 18.88 months)	-	Junior or below = 26.3%Senior = 55.8%College or above = 17.9%	17–29 (22.08)	-	43.2%	37.9%	5.3%	-	-	4
Qi SZ, 2006 [19]	Not reported	3 cities in 2 provinces	95	MSM venues	RDS	-	-	-	17–29 (22.08)	-	-	-	5.3%	-	-	4
Qiu DH, 2008 [47]	12/2007–05/2008	Not reported	21	Not reported	Not reported	-	-	-	20–35 (25.1)	61.9%	-	-	-	-	-	3
Shi XD, 2010 [48]	2009	Shenzhen (Guangdong)	505	Peer referral, MSM venues	RDS	-	-	Junior or below = 33.7%Senior = 48.5%College or above = 17.8%	16–51 (23.48)	83.6%	-	-	7.9%	B = 34.3%H = 22.0%G = 35.3%O = 8.5%	-	4
Tao XR, 2010 [49]	08/2007–12/2008	Not reported	118	Peer-referral, MSM venues	Purpose	-	Student = 23.7%; Currently employed = 28.0% unemployed = 11.0%;	Junior or below = 18.6%Senior = 34.7%College or above = 46.6%	N/A (23)	16.1%	-	8.5%	5.9%	B = 36.4%H = 1.7%G = 51.7%O = 10.2%	-	4
Tao XR, 2010 [5]	08/2007–12/2008	Not reported	118	MSM venues; peer referral	RDS; pilot	-	-	-	N/A (23)	-	-	-	-	-	I = 27.8%R = 11.4%Both = 60.8%	5
Tao ZM, 2010 [50]	2009	Chengdu (Sichuan)	205	Not reported	Not reported	-	-	-	-	-	-	-	-	-	-	3
Wang LL, 2008 [51]	10/2007	Harbin (Heilongjiang)	152	MSM venues; peer referral	Cluster sampling	-	Student = 10.5%; Currently employment = 86.2%; Unemployed = 2.0%	Junior or below = 23.7%%Senior = 45.4%College or above = 30.9%	18–39	-	-	-	10.5%	B = 19.7%H = 0.7%G = 76.3%O = 10.5%	-	5
Wong FY, 2008 [4]	04/2006–06/2006	Shanghai	239	Peer referral	Snowball; RDS	-	-	-	25.2±4.7	-	-	-	-	-	-	5
Xi SJ, 2009 [22]	08/2008–11/2008	Hangzhou (Zhejiang)	206	MSM venues	Convenience	-	-	Junior or below = 13.1%Senior = 74.8%College or above = 12.1%	17–29 (22.6±2.5)	96.1%	-	-	3.9%	B = 4.4%H = 0.5%G = 76.7%O = 18.4%	-	4
Xu J, 2011[52]	2006–2008	Chongqing (Sichuan)	172	MSM venues	Snowball	-	-	Junior or below = 42.4%Senior = 40.7%College or above = 16.9%	-	44.9%	-	-	-	B = 34.3%H = 5.2%G = 56.4%O = 4.1%	-	5
Yuan F, 2011 [53]	03/2008–12/2008	Chengdu (Sichuan)	120	MSM venues	Snowball	-	-	Junior or below = 36.7%Senior = 40.8%College or above = 21.7%	20–33	-	-	-	-	-	-	4
Zhang BU, 2004 [54]	2001	Not reported	57	Not reported	Target sampling	-	-	Junior or below = 24.6%Senior = 40.4%College or above = 35.1%	≥17	-	-	2.1%	-	B = 19.3%H = 7.0%G = 56.1%O = 17.5%	-	4
Zhang QP, 2011 [23]	2009	Chongqing (Sichuan)	190	Community outreach; MSM venues	Snowball	-	Student = 6.3%; Currently employment = 80.5%; Unemployed = 8.9%	Junior or below = 42.1%Senior = 41.1%College or above = 16.8%	16–47 (24.2±6)	59.5%	-	13.2%	10.5%	G = 56.8%	-	6
Zhuang T, 2005 [55]	02/2004–07/2004	Not reported	69	Peer referral	Target sampling	-	-	Junior or below = 14.5%Senior = 50.7%College or above = 31.9%	17–28 (21.35)	-	-	18.8%	4.1%	-	-	3

φ MSM venues include bars, clubs, saunas, bathhouses and massage parlors where MSM congregate.

† RDS: Respondent Driven sampling.

* B: Bisexual; Hetero: Heterosexual; G: Gay/homosexual; O: Others.

‡ I: Insertive; R: Receptive.

**Table 2:** Studies which reported the sexual behaviours among money boys in China.

First author, published year	Study Period	Study location	Sample size (N)	Recruitment methodsφ	Sampling methods†	Type of partners*	Number of sexual partners	Number of sexual acts∧	Type of sex	Condom use at last sex act (n/N)¶	Consistent condom use in past six months (n/N)¶	Quality assessment score
Cai YM, 2008 [37]	08/2004	Shenzhen (Guangdong)	138	Community	RDS	M_com_	-	1–3/wk = 71%; 4–6/wk = 11.6%; 7–9/wk = 2.2%; >10/wk = 10.9%; unspecified = 4.3%	Anal	-	56/138	4
						F_com_	1–3 = 75%; >10 = 2.8%; not reported = 22.2%	-	Vaginal	-	12/27	
Chen B, 2007 [39]	06/2007–10/2007	Chengdu (Sichuan)	42	Community outreach; MSM venues	Purpose	M_Any_	-	-	Anal	16/42	40/42	3
Cheng WB, 2010 [21]	05/2009–08/2009	Guangzhou (Guangdong)	151	Peer-referral, MSM venues	Purpose	M_Any_	-	-	Anal	-	47/151	6
						M_Com_	-	-	Anal	-	80/151	
						M_Com_	-	-	Oral	-	22/151	
Deng YH, 2009 [41]	11/2008–12/2008	Kunming (Yunnan)	45	Peer referral	Not reported	M_Any_	-	-	Anal	26/45	-	3
						M_Com_	-	-	Anal	45/45	-	
Li Y, 2010 [56]	2004	Multiple locations (9 cities)	264	Peer referral	Snowball	M_Com_	-	-	Oral	71/199	79/206	4
						M_Com_	-	-	Anal	175/203	173/207	
Liu HJ, 2008 [6]	11/2007–12/2007	Shenzhen (Guangdong)	58	Peer-referral, MSM venues	RDS	M_Any_	-	-	Anal	-	38/56	4
						M_Com_	-	-	Anal	-	6/9	
						F_Any_	-	-	Vaginal	-	9/25	
Liu S, 2011 [1]	06/2009–10/2009	Shenzhen (Guangdong)	418	MSM venues	Time-location cluster sampling	M_Cas_	-	-	Anal	-	117/168	7
						M_reg_	-	-	Anal	-	85/134	
						M_com_	-	-	Anal	-	341/414	
						F_Cas_	-	-	Vaginal	-	78/133	
						F_Reg_	-	-	Vaginal	-	75/171	
Liu ZQ, 2009 [45]	03/2007–06/2007	Tianjin	89	MSM venues	Not reported	M_Any_	<10 = 50.6% ; ≥10 = 49.4%	-	Anal	80/89	66/89	4
Meng XD, 2010 [9]	10/2008–11/2008	Changchun (Jilin)	86	Not reported	Convenience	M_Com_	-	-	Anal	64/239	-	3
Qi SZ, 2006 [19]	Not reported	3 cities in 2 provinces	95	MSM venues	RDS	M_Any_	-	-	Anal	64/95	-	4
Shi XD, 2010 [48]	2009	Shenzhen (Guangdong)	505	Peer referral, MSM venues	RDS	M_Com_	-	-	Anal	477/505	-	4
Tao XR, 2010 [49]	08/2007–12/2008	Not reported	118	Peer-referral, MSM venues	Purpose	M_Any_	-	-	Anal	-	49/118	4
						M_Com_	-	-	Anal	84/118	-	
						F_Any_	Mean = 9	-	Vaginal	70/118	26/118	
Wang LL, 2008 [51]	10/2007	Harbin (Heilongjiang)	152	MSM venues; peer referral	Cluster sampling	M_Any_	-	-	Anal	84/152	-	5
						M_Com_	-	-	Anal	53/111	-	
						F_Any_	-	-	Vaginal	26/41	-	
Xi CJ, 2009 [22]	08/2008–11/2008	Hangzhou (Zhejiang)	206	MSM venues	Convenience	M_Com_	-	-	Anal	154/206	98/206	4
Xu J, 2011 [52]	2006	Chongqing (Sichuan)	47	MSM venues	Snowball	M_Any_	-	-	Anal	31/47	23/47	5
						M_Com_	-	(P6M) <5 = 74.4%, ≥5 = 25.6%	Anal	33/27	27/47	
Xu J, 2011 [52]	2007	Chongqing (Sichuan)	71	MSM venues	Snowball	M_Any_	-	-	Anal	53/71	39/71	5
						M_Com_	-	(P6M) <5 = 52.1%, ≥5 = 47.9%	Anal	58/71	42/71	
Xu J, 2011 [52]	2008	Chongqing (Sichuan)	54	MSM venues	Snowball	M_Any_	-	-	Anal	41/54	14/54	5
						M_Com_	-	(P6M) <5 = 50%, ≥5 = 50%	Anal	43/54	27/54	
Yuan F, 2011 [53]	03/2008–12/2008	Chengdu (Sichuan)	120	MSM venues	Snowball	M_Any_	-	-	Anal	89/117	-	4
Zhang BU, 2004 [54]	2001	Not reported	57	Not reported	Target sampling	M_Any_	-	-	Anal	25/42	-	4
						M_Any_	-	-	Oral	9/42	-	
Zhang QP, 2011 [23]	2009	Chongqing (Sichuan)	190	Community outreach; MSM venues	Snowball	M_Any_	-	-	Anal	140/188	85/188	6
						F_Any_	-	-	Vaginal	22/63	18/63	
Zhuang T, 2005 [55]	02/2004–07/2004	Not reported	69	Peer referral	Target sampling	M_Any_	-	-	Anal	16/69	-	3

φ MSM venues include bars, clubs, saunas, bathhouses and massage parlors where MSM congregate.

† RDS = Respondent Driven sampling.

* M_Any_ = Any types of male sexual partners; M_Reg_ = Regular male sexual partners; M_Cas_ = Casual male sexual partners; M_Com_ = Commercial male sexual partners (i.e. male clients); F_Any_ = Any types of female sexual partners; F_Reg = _Regular female sexual partners; F_Cas_ = Casual female sexual partners; F_Com_ = Commercial female sexual partners (i.e. female clients);

∧ wk = week(s); P6M = past six months.

¶ condom use = number of money boys who used condom/number of money boys who had sex during the duration.

**Table 3:** Studies which reported sexually transmitted infections and HIV prevalence among money boys in China.

First author, published year	Study Period	Study location	Sample size (N)	Recruitment methods*	Sampling methods†	Types of Infectious Diseases∧	Laboratory test¶	Prevalence n (%)	Quality assessment score
Cheng WB, 2010 [21]	05/2009–08/2009	Guangzhou (Guangdong)	151	Peer-referral, MSM venues	Purpose	HIV	Not reported	17 (11.3%)	6
						Syphilis	Presumptive: TPPA; Confirmatory: RPR	30 (19.9%)	
Li QH, 2006 [42]	01/2005–12/2005	Beijing	85	Clinics	Convenience	HIV	Presumptive: RPR	5 (5.9%)	4
						Syphilis	Presumptive: TPPA; Confirmatory: RPR	17 (20.0%)	
						HSV	Not reported	7 (8.2%)	
Li YF, 2012 [44]	03/2011–10/2011	Jining (Shandong)	41	Sentinel sites, VCT clinics and peer referral	Not reported	HIV	Presumptive: RPR; Confirmatory: ELISA	3(7.3%)	5
Liu S, 2011 [1]	06/2009–10/2009	Shenzhen (Guangdong)	418	MSM venues	Time-location cluster sampling	HIV	Presumptive: ELISA; Confirmatory: WB	14 (3.4%)	7
						Syphilis	Presumptive: TPPA; Confirmatory: RPR	44 (10.5%)	
						HSV-2	ELISA	46 (11.0%)	
						HIV/Syphilis co-infection	Not reported	8 (1.9%)	
Liu ZQ, 2009 [45]	03/2007–06/2007	Tianjin	89	MSM venues	Not reported	HIV	Confirmatory: WB; Presumptive: ELISA	6 (6.7%)	4
						Syphilis	Confirmatory: RPR	18 (20.2%)	
Qi SZ, 2006 [19]	Not reported	3 cities in 2 provinces	95	MSM venues	RDS	HIV	Not reported	0 (0.0%)	4
						Syphilis	Presumptive: TPPA; Confirmatory: RPR	16 (19.5%)	
						HSV	Not reported	5 (6.1%)	
Qi SZ, 2006 [46]	Not reported	3 cities in 2 provinces	82#	MSM venues	RDS	Gonorrhoea	PCR	8 (8.4%)	4
						Chlamydia	PCR	12 (12.1%)	
						Gonorrhoea/Chlamydia co-infection	PCR	2 (2.1%)	
Qiu DH, 2008 [47]	12/2007–05/2008	Not reported	16#	Not reported	Not reported	HIV	Confirmatory: WB	2 (12.5%)	3
						Syphilis	Presumptive: TPPA; Confirmatory: TRUST	2 (12.5%)	
						HIV/Syphilis co-infection	Not reported	1 (6.3%)	
Shi XD, 2010 [48]	2009	Shenzhen (Guangdong)	505	Peer referral, MSM venues	RDS	HIV	Presumptive: ELISA; Confirmatory: WB	18 (3.6%)	4
						Syphilis	Presumptive: TPPA; Confirmatory: RPR	56 (11.1%)	
						HCV	ELISA	6 (1.2%)	
Tao XR, 2010 [49]	08/2007–12/2008	Not reported	118	Peer-referral, MSM venues	Purpose	HIV	Presumptive: RPR	6 (5.1%)	4
						Syphilis	Not reported	12 (10.2%)	
Tao ZM, 2010 [50]	2009	Chengdu (Sichuan)	205	Not reported	Not reported	HIV	Not reported	1 (0.5%)	3
						Syphilis	Not reported	10 (4.9%)	
Xu J, 2011 [52]	2006	Chongqing (Sichuan)	47	MSM venues	Snowball	HIV	Presumptive: ELISA; Confirmatory: WB	6 (12.8%)	5
	2006	Chongqing (Sichuan)	47	MSM venues	Snowball	Syphilis	Presumptive: RPR; Confirmatory: TPPA	9 (19.1%)	
	2007	Chongqing (Sichuan)	71	MSM venues	Snowball	HIV	Presumptive: ELISA; Confirmatory: WB	7 (9.9%)	
	2007	Chongqing (Sichuan)	71	MSM venues	Snowball	Syphilis	Presumptive: RPR; Confirmatory: TPPA	6 (8.5%)	
	2008	Chongqing (Sichuan)	54	MSM venues	Snowball	HIV	Presumptive: ELISA; Confirmatory: WB	4 (7.7%)	
	2008	Chongqing (Sichuan)	54	MSM venues	Snowball	Syphilis	Presumptive: RPR; Confirmatory: TPPA	3 (5.6%)	
Yuan F, 2011 [53]	03/2008–12/2008	Chengdu (Sichuan)	120	MSM venues	Snowball	HIV	Confirmatory: WB	5 (4.2%)	4
						Syphilis	Presumptive: RPR	14 (11.7%)	
						HCV	Not reported	7 (5.9%)	
						HBV	Not reported	5 (4.2%)	
Zhang QP, 2011 [23]	2009	Chongqing (Sichuan)	190	Community outreach; MSM venues	Snowball	HIV	Presumptive: RPR and ELISA; Confirmatory: WB	21 (11.1%)	6
						Syphilis	Presumptive: TPPA; Confirmatory: RPR	22 (11.6%)	
						HIV/Syphilis co-infection	Not reported	6 (3.2%)	
Zhuang T, 2005 [55]	02/2004–07/2004	Not reported	86#	Peer referral	Target sampling	HIV	Not reported	0 (0.0%)	3
						Syphilis	Not reported	5 (5.8%)	

# Number of people who were voluntary provided blood sample for HIV/STI testing, this number is not the same as the total sample size (number of participates recruited in the study).

* MSM venues include bars, clubs, saunas, bathhouses and massage parlors where MSM congregate.

† RDS: Respondent Driven sampling.

∧ HBV: Hepatitis B virus; HCV: Hepatitis C virus; HIV: Human immunodeficiency virus; HSV-2: Herpes simplex virus 2.

¶ EIA: Enzyme immunoassay; ELISA: Enzyme-linked immunosorbent assay; RPR: Rapid Plasma Reagin; TPPA: Treponema pallidum Particle Agglutination assay; TRUST: Toluidine red unheated serum test.

### Statistical analysis

Meta-analyses were carried out by using the Comprehensive Meta-Analysis software (V2.0, Biostat, Englewood, New Jersey) for indicators with 3 or more studies [11]. We calculated the mean and 95% confidence intervals (CI) based on a binomial distribution for the study variables with less than 3 studies. Heterogeneity tests across studies were detected by the Cochran Q-test (*p<*0.10 represents statistically significant heterogeneity) and *I* ^2^ statistic [12]–[14]. If high and significant heterogeneities were detected across studies, a random-effect model was used to calculate the effect rate of pooled prevalence estimates and 95% CI [15]. A fixed-effect model was used when low heterogeneities were observed across studies. Sampling sizes of the study were also taken into account in both models. We investigated factors related to heterogeneity among studies by using meta-regression [16]. Potential study characteristics associated with high heterogeneity were examined in a multiple variables meta-regression model. The multiple variables used in this study were the language of publication, size of study, recruitment method, sampling method and study time period. The regression coefficient and *p* values (*p*<0.10 indicates that the factor is significantly associated with heterogeneity) for each study characteristic on meta-analysis were reported. Potential presence of publication bias was measured by the Begg and Mazumdar rank correlation (*p<*0.05 represents statistically significant publication bias) [17], [18]. Odd ratio and 95% CI for study indicators were calculated to compare the risk between MBs and the broader MSM population.

## Results

### Flow of included studies

**Figure 1:**
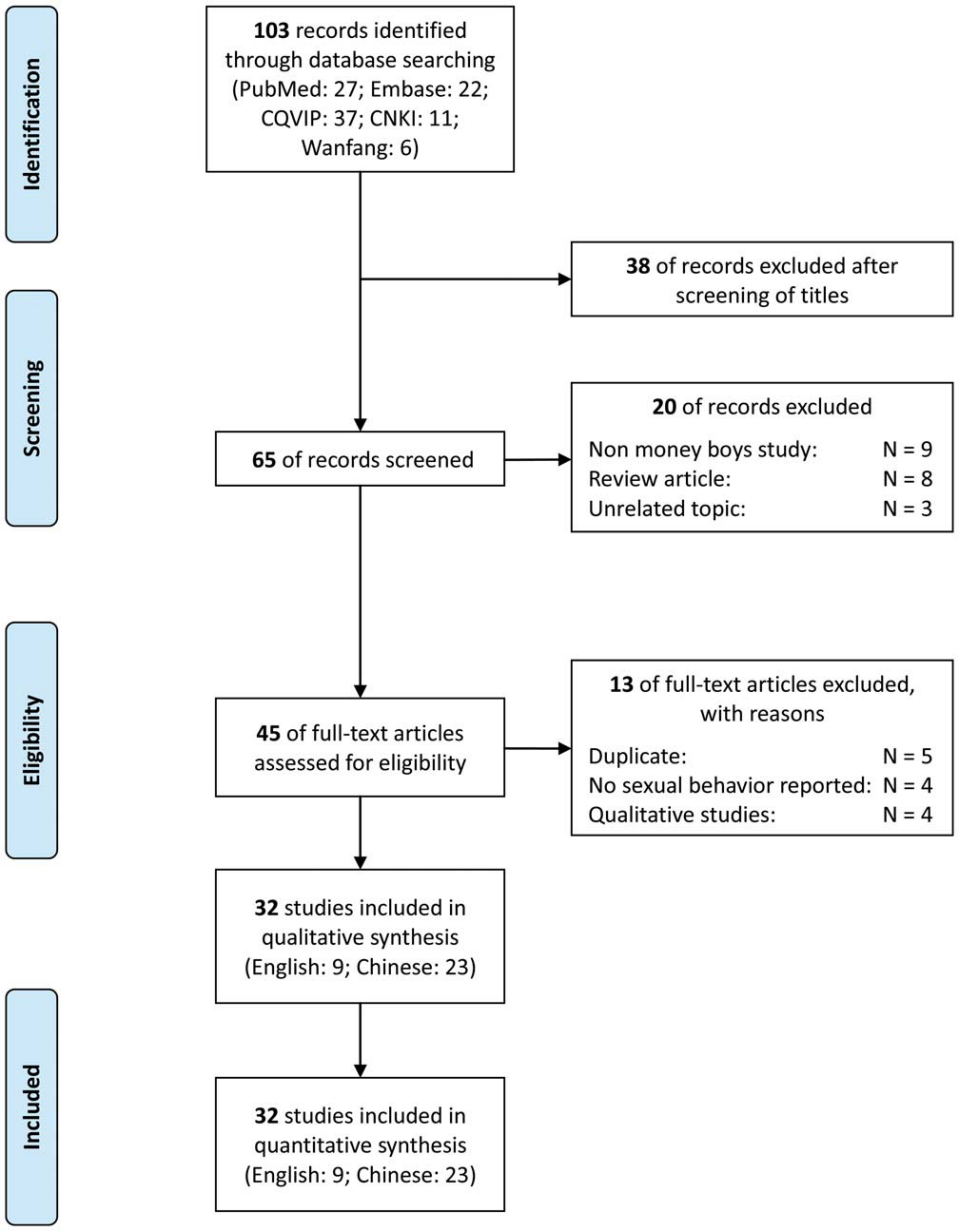
PRISMA flow chart for selection of studies with number of articles (N).

We identified 103 records related to the topic from four electron databases. Due to duplication of records across the databases and unrelated topics, we excluded 38 articles after screening the titles. We screened the abstracts of the remaining 65 articles, of which 20 articles were excluded. The remaining 45 articles were eligible for full-text screening, 13 articles were further excluded due to duplications of repeated studies and the nature of non-quantitative studies. Finally, 32 articles (9 in English and 23 in Chinese) were included in this data synthesis (Figure 1).

### Study characteristics

The sample size of eligible studies ranged from 15 to 505 (median: 118.0; IQR: 57.3–203.5). Studies were conducted from 2001 to 2011. The mean age of the participants across the studies was 23.1 years (range: 16–47 years). All studies were cross-sectional studies. Half of the studies (16/32) recruited MBs from MSM venues such as gay bars and saunas.

### Demographic characteristics

Demographic characteristics of MBs were shown in Table 4. Pooled estimates across all studies indicated that the average years of selling sex was approximately 1 to 2 years [1], [3], [19]–[21]. A substantial proportion of the study participants were unemployed (6.9%, 95% CI: 3.3–13.9%), senior-high school graduates (68.5%, 95% CI: 64.0–72.6%) and rural-to-urban migrants (67.5%, 95% CI: 46.1–83.5%). Overall, 8.7% (95% CI: 5.6–13.5%) of MBs reported to be currently married to a female. The majority of MBs (53.8%, 95% CI: 45.3–62.0%) self-identified as homosexual, while 30.2% (24.3–36.9%) identified as bisexual and only 6.2% (95% CI: 3.5–10.6%) identified as heterosexual; the remaining 8.9% did not indicate their sexual orientation. Approximately half of the MBs (49.8%, 95% CI: 43.9–55.8%) reported engaging in both insertive and receptive sexual positioning. The majority (73.8%) of MBs sought for their male clients from MSM venues such as bars, clubs and bathhouses; while one-fourth found clients on the Internet [22]. In comparison with the broader MSM population, MBs were less likely to be married (OR = 0.29; 95% CI: 0.24–0.34) and unemployed (OR = 0.22; 95% CI: 0.17–0.28) but highly mobile (OR = 4.87; 95% CI: 4.34–5.46) and with poorer literacy level (OR = 0.35; 95% CI: 0.31–0.40).

**Table 4:** Comparison between Chinese money boys and men who have sex with men in their demographic characteristics, HIV-related behaviours and HIV/STIs testing rates and burden of infectious diseases.

Study characteristics	Money boys		Men who have sex with men		Odd Ratios (95% CI)*
	Pooled estimate % (95% CI)∧	No. of studies (*n*)	Pooled estimate % (95% CI)	Reference Source	
**I. Demographic characteristics**					
Migrants	67.5% (46.1–83.5%)	9	29.9% (29.0–30.7%)^f^	[57]	4.87 (4.34–5.46)*
Current married	8.7% (5.6–13.5%)	18	24.7% (23.0–26.5%)^c^	[27]	0.29 (0.24–0.34)*
Occupation					
*Unemployed*	6.9% (3.3–13.9%)	3	31.1% (29.2–33.1%)^c^	[27]	0.22 (0.17–0.28)*
Literacy level					
*Senior high school or above*	68.5% (64.0–72.6%)	21	86.1% (84.6–87.5%)^c^	[27]	0.35 (0.31–0.40)*
**II. HIV-Related behaviours**					
Self-identified sexual orientation					
*Bisexual*	30.2% (24.3–36.9%)	12	38.0% (36.0–40.0%)^c^	[27]	0.71 (0.62–0.80)*
*Homosexual*	53.8% (45.3–62.0%)	17	52.0% (49.9–54.0)^c^	[27]	1.08 (0.96–1.20)
*Heterosexual*	6.2% (3.5–10.6%)	12	9.1% (8.0–10.4%)^c^	[27]	0.60 (0.53–0.83)*
*Others*	8.9% (6.2–12.4%)	11	0.9% (0.6–1.4%)^c^	[27]	10.76 (6.77–17.10)*
Preferable sexual positioning					
*Insertive*	24.5% (19.8–30.0%)#	2	43.8% (41.7–45.9%)^c^	[27]	0.42 (0.31–0.56)*
*Receptive*	24.2% (19.4–29.6%)#	2	13.1% (11.8–14.6%)^c^	[27]	2.12 (1.56–2.87)*
*Both*	49.8% (43.9–55.8%)#	2	N/A		
Ever used drug	14.8% (10.6–20.3%)	11	8.3% (7.2–9.5%)^c^	[27]	1.92 (1.54–2.39)*
Participated in group sex in the past 12 months	40.9% (34.5–47.7%)	2	18.6% (17.0–20.3%)^c^	[27]	3.03 (2.25–4.07)*
Condom Usage†					
*With any male partners*	LA: 69.2% (59.1–77.7%)	12	LA: 61.6% (58.4–64.8%)^f^	[35]	2.39 (2.07–2.76)*
	P6M: 48.5% (37.7–59.6%)	8	P6M: 36.3% (33.7–38.9%)^f^		1.65 (1.42–1.93)*
*With regular male partners*	P6M: 63.4% (55.0–71.1%)^s^	1	19.9% (14.1–27.2%)^b^	[35]	6.97 (4.76–10.22)*
*With casual male partners*	P6M: 69.6% (62.3–76.1%)^s^	1	30.4% (20.5–42.5%)^b^	[35]	5.24 (3.73–7.37)*
*With male clients*	LA: 79.4% (62.4–89.9%)	11	N/A	N/A	N/A
	P6M: 61.3% (47.2–73.6%)	9			
*With commercial male sex workers*	N/A	N/A	LA: 75.2% (58.2–86.9%)^f^	[35]	N/A
			P6M: 58.0% (51.2–64.5%)^f^		
*With any female partners*	LA: 52.5% (35.6–68.8%)	3	LA: 41.4% (35.5–47.5%)^e^	[32]	1.56 (1.18–2.08)*
	P6M: 26.0% (20.4–32.5%)	3	P6M: 25.6% (23.0–28.4%)^a^		1.02 (0.74–1.40)
*With regular female partners*	P6M: 43.9% (36.6–51.4%)^s^	1	P6M: 23.3% (11.2–42.1%)^e^	[32]	2.58 (1.57–4.22)*
*With casual female partners*	P6M: 58.6% (50.1–66.7%)^s^	1	P6M: 39.0% (28.8–50.3%)^e^	[32]	2.21 (1.25–3.93)*
*With female clients*	LA: 75.7% (68.8–81.5%)^s^	1	N/A	N/A	N/A
	P6M: 44.4% (27.2–63.1%)^s^	1			
*With female sex workers*	N/A	N/A	LA: 80.3% (60.4–91.6%)^d^	[32]	N/A
			P6M: 55.8% (41.4–69.4%)^e^		
**III. HIV/STIs Testing Rates**					
*Ever tested for HIV*	44.5% (29.7–60.3%)	8	51.2% (39.0–63.4%)^h^	[30]	0.76 (0.67–0.88)*
*Tested for HIV in the past 12 months*	31.7% (21.3–44.5%)	8	43.7% (37.1–50.2%)^h^	[30]	0.59 (0.52–0.70)*
*Ever tested for STIs*	40.5% (35.6–45.8%)#	2	N/A	N/A	N/A
**IV. Diseases Prevalence**					
*HIV*	6.0% (4.2–8.5%)	16	4.7% (3.9–5.6%)^g^	[58]	1.29 (1.09–1.54)*
*Syphilis*	12.4% (9.9–15.3%)	15	13.5% (11.8–15.3%)^g^	[58]	0.91 (0.79–1.04)
*HIV-syphilis co-infection*	2.2% (1.1–4.1%)	3	2.7% (1.8–4.0%)^g^	[58]	0.81 (0.46–1.43)

† LA: condom use at last sex act; P6M: consistent condom use in the past 6 months.

∧ Meta-analysis was not performed for study variables less than three studies.

* Odds ratio is statistically significant at the 5% level (*p<*0.05).

# Weighted mean and 95% confidence intervals for indicators less than three studies.

s 95% confidence intervals were estimated by a bionomical distribution from one study.

a Study period 2002–2008.

b Study period 2003–2007.

c Study period 2005–2006.

d Study period 2005–2007.

e Study period 2005–2008.

f Study period 2006–2008.

g Study period 2007–2008.

h Study period 2009.

### HIV-related risk behaviour

A relatively high percentage of MBs (40.9%, 95% CI: 34.5–47.7%, *n* = 2) had participated in group sex in the past 12 months and 14.8% (95% CI: 10.6–20.3%) had ever used drugs (Table 4), indicating a greater likelihood of participating in group sex (OR = 3.03; 95% CI: 2.25–4.07) and using drug (OR = 1.92; 95% CI: 1.54–2.39) in comparison with the broader MSM population. It was estimated that 69.2% (95% CI: 59.1–77.7%) of MBs used a condom with their male sex partners in their last anal sex act, while less than one-third of MBs (30.5%, 95% CI: 21.4–41.4%) used a condom in their last oral sex act. Furthermore, our results showed that 79.4% (95% CI: 62.4–89.9%) of MBs used a condom with their male clients at last anal act and 61.3% (95% CI: 47.2–73.6%) reported consistent condom use in the past 6 months. In contrast, condom use levels were lower with female clients in the last sex act (75.7%, *n* = 1) and in the past 6 months (44.4%, *n* = 1). Similarly, there were lower levels of condom use in regular and non-commercial causal partnerships between MBs and females than between MBs and other males. Consistent condom use in the past six months between MBs and their male regular partners was 63.4% (*n* = 1) and consistent condom use with non-commercial male causal partners was 69.6% (*n* = 1) compared with their lower levels with regular female partners (43.9%, *n* = 1) and non-commercial female casual partners (58.6%, *n* = 1). In comparison with other MSMs, MBs were more likely to have consistent condom use with their regular male (OR = 6.97; 95% CI: 4.76–10.22), casual male (OR = 5.24; 95% CI: 3.73–7.37), regular female (OR = 2.58; 95% CI: 1.57–4.22) and non-commercial casual female partners (OR = 2.21; 95% CI: 1.25–3.93) in the past six months.

### HIV/STI testing rate

Eight studies reported rates of ever testing for HIV and nine studies HIV testing rate in the past 12 months but only 2 reported the percentage of MBs who had ever been tested for any sexually transmitted diseases. The pooled estimate of the proportion of MBs who had ever tested for HIV from 2001 to 2008 was 44.5% (95% CI: 29.7–60.3%) while the HIV testing rate in the past 12 months was 31.7% (95% CI: 21.3–44.5%) during the period 2001–2011 (Table 4); and there was no association between testing rates and study period (linear regression trend test, *p_trend_* = 0.075 and 0.094 for ever-tested and past-12-month respectively). Our results showed that MBs were less likely to test for HIV in their lifetime (OR = 0.76; 95% CI: 0.67–0.88) and in the past 12 months (OR = 0.59; 95% CI: 0.52–0.70) than the broader MSM population. About 40.5% (95% CI: 35.6–45.8%) had tested for STIs in their lifetime.

### Epidemic trends of HIV, STIs and co-infection

Out of 32 studies, 16 reported the prevalence of HIV/STI among MBs in China. According to our pooled prevalence estimate from meta-analysis, the HIV prevalence (*n* = 16) among MBs in China was 6.0% (95% CI: 4.2%–8.5%). Studies indicated that the HIV prevalence among MBs peaked as high as 11.3% in Guangzhou [21] and 11.1% in Chongqing [23]. Prevalence of syphilis (*n* = 15) was 2-times fold higher at 12.4% (95% CI: 9.9–15.3%) than the HIV prevalence among MBs. A similar proportion (10.1%, 95% CI: 7.9–12.8%) of MBs were currently infected with HSV-2 (*n* = 3). The prevalence of HIV-syphilis co-infection (*n* = 3) among MBs was 2.2% (95% CI: 1.1–4.1%). The seroprevalence of *Chlamydia trachomatis* (*n* = 1) and *Neisseria gonorrhoea* (*n* = 1) infection was 12.6% (95% CI: 7.3–20.9%) and 8.4% (95% CI: 4.3–15.9%), respectively. Further, we estimated that about 4.2% (95% CI: 1.7–9.6%) and 2.7% (95% CI: 0.5–12.0%) of MBs had HBsAg (*n* = 1) and HCV-Ab (*n* = 2) detected in their serum. MBs were 1.29 (95% CI: 1.09–1.54) times more likely to be infected with HIV than the broader MSMs population there were no significant differences in the odds of syphilis infection (OR = 0.91; 95% CI: 0.79–1.04) and HIV-syphilis co-infection (0.81; 95% CI: 0.46–1.43) between the two populations (Table 4).

### Publication bias and heterogeneities

Among all the sub-group analyses among MBs, potential publication biases were only detected in reporting level of literacy (*p = *0.046) and ever tested for HIV (*p = *0.026). Out of twenty subgroups meta-analyses, fifteen were found to have high heterogeneities (Table S3). No significant association was detected between study design and the high heterogeneity in any subgroup meta-analyses. Study year (early 2000s *versus* late 2000s) was one of the factors associated with high heterogeneities in condom use with male clients at last sex act (b = −0.234, *p*<0.001). Language of study (Chinese *versus* English) was significant in reporting the percentage of migrant (b = −1.694, *p*<0.001) and condom use with male clients in the past 6 months (b = 0.509, *p*<0.001). Furthermore, sample size (≥100 *versus* <100) was significant in reporting the percentage of MBs who were married (b = −1.129, *p* = 0.095), condom use with male clients at last sex act (b = 1.061, *p*<0.001) and in the past 6 months (b = −0.545, *p*<0.001). Study method (non-snowball *versus* snowball sampling) was significantly associated in heterogeneity in reporting the proportion of migrant (b = −0.516, *p*<0.001), self-reported heterosexual orientation (b = 0.745, *p* = 0.066), condom use with male clients at last sex act (b = −0.982, *p*<0.001) and in the past 6 months (b = 0.687, *p*<0.001) (Table S4).

## Discussion

This is the first comprehensive systematic review on the demographical, behavioural and HIV/STI epidemiological data of MBs in China. The pooled evidence across available studies indicates that Chinese MBs are generally unmarried, employed, and at low literacy level than the broader MSM population. Additionally, they are also highly mobile due to their common migratory background. This indicates a lower accessibility to healthcare, job opportunities and remunerations than other urban residents due to their lacking of legitimate residential status under the Chinese household registration system [24].

Overall, the risk of HIV infection among MBs is significantly greater than among the broader MSM population. This is a result of multiple factors. Condom usage with all types of sexual partnerships of Chinese MBs are substantially greater than the broader MSM population, even in regular and non-commercial casual partnerships (OR = 6.97; 95% CI: 4.76–10.22 and OR = 5.24; 95% CI: 3.73–7.37, Table 4). This may be due to their self-perception of higher risk of HIV infection related to their occupational practices. Past studies showed that although MBs have lower levels of literacy they usually have greater awareness of risks of HIV infection than the broader MSM population. Hence, they are more likely to have safe sex in any type of sexual partnership as well as persuade their clients to use condom during the sex trade [3], [9], [25]. However, the protective effects of condom usage are likely out-weighted by their greater numbers of risk events. First, the proportion of MBs taking a receptive role during intercourse is twice of the broader MSM population, and the risk of HIV infection while taking the receptive role is known to be approximately 10 times greater than the risk associated with the insertive role [26]. Second, MBs are more likely to have multiple sexual partners than the broader MSM population, as approximately 40.9% of MBs have participated in group sex in the past 12 months compared with only 18.6% among other MSMs (OR = 3.03; 95% CI: 2.25–4.07) [27]. Third, drug usage is more frequent among MBs than the broader MSM population. An estimated 14.8% (95% CI: 10.6–20.3%) of MBs had ever used drugs but only 8.3% of MSM had used drugs (OR = 1.92; 95% CI: 1.54–2.39) [27]. Drug consumption is not only an indication of injecting drug behaviours and related transmission risks but also directly related to unsafe sex among Chinese MSM [28], [29]. Fourth, the substantially lower HIV testing rate in the past 12 months among MBs (31.7% compared with 43.7% among MSM) implies lower awareness of their own HIV infection status which may in turn contribute to more risk behaviours [30]. As a previous study estimated that up to 87% of HIV cases among MSM in China remained undiagnosed [31], the proportion may be even higher among Chinese MBs.

Bisexual behaviours are generally less common among MBs than other MSMs in China. However, over one-third (36.4%) of Chinese MBs identified themselves as bisexual/heterosexual, suggesting the likelihood of sexual exposure of MBs with females remain substantial. The fact that only 8.7% of Chinese MBs are currently married to a female, compared with 24.7% among the broader MSM population, is likely due to the younger age among MB participants (mean age: 23.1 years old). Higher marriage rates are observed among older MBs [32]. Although the rates of condom use with both regular and casual female partners among MBs are significantly greater than condom use among other MSMs, as MBs often engage in group sex and multiple partnerships with both male and female, their potential role as a bridge of HIV transmission in this triangle of population groups cannot be neglected.

Several limitations in this study should be noted. First, the sample sizes of the studies are generally small. Approximately 72% of the studies (23/32) had sample sizes less than 200 and most of them were conducted in single urban cities, which may reduce the generalisability of the results nationwide. Second, all eligible articles included in this review are cross-sectional studies. Cohort studies are often a better design for understanding the changing behaviours and epidemic trends, but were not available. Third, pooled analyses stratified by different sampling methods were not conducted due to the limited number of studies available in each meta-analysis. Fourth, only very few studies reported the prevalence of STIs (Chlamydia, gonorrhoea, HBV, HCV and HSV); the lower seroprevalence of HBV among MBs (4.2%) than the general adult Chinese population (8–11.0%) [33], [34] is likely an indication of sampling and publication biases. Furthermore, some studies did not report the methods of the laboratory test for the infectious diseases, which may lead to uncertainties in the reliability and validity of the measurement. Fifth, it has been reported that both HIV disease burden and risk-behaviours among MSM vary geographically and temporally [32], [35], but there were insufficient studies available for investigations among Chinese MBs. Sixth, high heterogeneities were observed in our subgroup meta-analyses, we have identified several factors which may contribute to heterogeneities but detailed subgroups analyses were not possible due to limited availability of other potential underlying factors.

Money boys represent a unique sub-population of MSM and a core group for HIV/STI transmission from the gay community to the broader populations. HIV/STI prevention and sexual health promotion are essential for Chinese MBs. First, due to the commercial nature of their sexual acts, condom distribution in male commercial sex venues should be further scaled up. Since a substantial proportion of MBs engaged in bisexual activities, HIV intervention strategies should also target both male and female clients of MBs at identifiable gay venues. Peer-support, counselling, HIV prevention and education are necessary for married MBs and their female partners. Second, the majority of MBs are internal migrants. Rural-to-urban migrants do not have equal access to employment as non-migrants. Social welfare networks should acknowledge that a disproportionate number of migrants enter the commercial sex industry and appropriate support systems should be implemented. Third, HIV/AIDS intervention strategies should also address the high mobility of MBs given their migration background, mobile voluntary HIV counselling and testing (VCT) sites should be set up to target MBs travelling between cities. Since approximately 3–18% of MSM who are tested for HIV do not receive their HIV results after screening [30], [35], free rapid HIV screening tests should be provided. Fourth, currently less than 3% (17/592) of HIV/AIDS national sentinel surveillance sites provide epidemiological surveillance for the MSM population [36]. Given the rapidly increasing HIV prevalence among Chinese MSM and even higher risk of HIV infection among MBs, it is therefore timely and important for the Chinese government to substantially scale-up the number of HIV/AIDS national sentinel surveillance sites specifically targeting MSM. Non-governmental and community-based organizations should work in partnership with the government to provide surveillance and health promotion at the grassroots level.

## Supporting Information

Table S1 **PRISMA checklist.**
(DOCX)

Table S2 **Quality Assessment of Cross Sectional Studies.**
(DOCX)

Table S3 **Publication bias and heterogeneity in subgroup meta-analyses.**
(DOCX)

Table S4 **Result of individual variable meta-regression models for each subgroup meta-analysis.**
(DOCX)
