# Supplementary material for: HIV and Sexually Transmissible Infections among Money Boys in China: A Data Synthesis and Meta-Analysis
Source: PLoS One. 2012 Nov 29;7(11):e48025. doi: 10.1371/journal.pone.0048025 (PMC3510224; doi:10.1371/journal.pone.0048025)
Supplement: Table S2 — Quality Assessment of Cross Sectional Studies. (DOCX) [file pone.0048025.s002.docx]

**Table S2. Quality Assessment of Cross Sectional Studies**

|  | **Question for Evaluating Prevalence Studies** | **Decision Criteria** | **Score** |
| --- | --- | --- | --- |
| Q1 | Was the population from which the sample was drawn clearly defined? | Yes: Background information, including: (i) study duration (e.g. yyyy/mm or yyyy) and (ii) recruitment locations (e.g. gay bars, saunas), was given clearly | 1 |
|  |  | No: Background information: neither (i) study duration or (ii) recruitment locations, was not given clearly | 0 |
| Q2 | Was the sampling method representative of the population intended to the study? | Yes: Probability sampling (including: simple random, systematic, stratified, cluster, two-stage and multi-stage sampling)was adopted | 1 |
|  |  | No: Non-probability sampling (including: purposive, quota, convenience and snowball sampling) was adopted | 0 |
| Q3 | Did the characteristics of respondents match the target population? | Yes: Inclusion/exclusion criteria in sample selection, including (i) age (e.g. 16 or older), (ii) duration of selling sex for money (e.g. self-reported to have sold sex for money within the previous 3 months) were identified clearly | 1 |
|  |  | No: Inclusion/exclusion criteria in sample selection: neither (i) age or (ii) duration of selling sex for money were not identified | 0 |
| Q4 | Was the response rate adequate? | Yes: Response rate was ≥ 80% (Rejection rate < 20%) | 1 |
|  |  | No: Response rate was not reported or < 80% | 0 |
| Q5 | Were the data collection methods standardised? | Yes: Identical methods of assessment and data collection were used to all respondents | 1 |
|  |  | No: Methods of assessment and data collection to all respondents were not identical | 0 |
| Q6 | Were measures shown to be reliable? | Yes: (i) Survey instrument: Survey was test-retested, piloted, adopted/adapted from other study (with reference) or tested by Cronbach’s alpha , (ii) STD's clinical tests: name(s) of screening/confirmation test was identified clearly | 1 |
|  |  | No: (i) Survey instrument: No information was identified in regards to the reliability of the instrument, (ii) STD's clinical tests: name of screening/confirmation test was not provided | 0 |
| Q7 | Were measures shown to be valid? | Yes: (i) Survey instrument: Study duration of condom usage (e.g. at last sex act, in the past 1 month) or frequency of using condom (e.g. Consistent/Always) were identified clearly, (ii) STD's clinical tests: name(s) of screening/confirmation test was identified clearly | 1 |
|  |  | No: (i) Survey instrument: Study duration or frequency of using condom were not provided, (ii) STD's clinical tests: name of screening/confirmation test was not provided | 0 |
| Q8 | Were the statistical methods appropriate? | Yes: Confident intervals or SD/variance were given for prevalence rate | 1 |
|  |  | No: Only the prevalence rate was given (CI or SD was not reported) | 0 |
