# Supplementary material for: HIV and Sexually Transmissible Infections among Money Boys in China: A Data Synthesis and Meta-Analysis
Source: PLoS One. 2012 Nov 29;7(11):e48025. doi: 10.1371/journal.pone.0048025 (PMC3510224; doi:10.1371/journal.pone.0048025)
Supplement: Table S3 — Publication bias and heterogeneity in subgroup meta-analyses. (DOCX) [file pone.0048025.s003.docx]

**Table S3. Publication bias and heterogeneity in subgroup meta-analyses**

| **Study characteristics** | **Number of studies (*n)*** | **Publication bias** | **Heterogeneity** | |
| --- | --- | --- | --- | --- |
|  |  | **Begg’s**  **(*p* values)** | ***I*^2^** | ***p-*value** |
| **I. Demographic characteristics** |  |  |  |  |
| Migrant | 9 | 0.835 | 97.60 | <0.001** |
| Current married | 18 | 0.910 | 92.03 | <0.001** |
| Occupation |  |  |  |  |
| *Unemployed* | 3 | 0.602 | 74.61 | 0.019* |
| Literacy level |  |  |  |  |
| *Senior high school or above* | 21 | 0.046* | 85.66 | <0.001** |
| **II. HIV-Related behaviours** |  |  |  |  |
| Sexual orientation |  |  |  |  |
| *Bisexual* | 12 | 0.131 | 90.10 | <0.001** |
| *Homosexual* | 17 | 0.217 | 93.75 | <0.001** |
| *Heterosexual* | 12 | 0.411 | 90.74 | <0.001** |
| *Others* | 11 | 0.186 | 79.26 | <0.001** |
| Preferable sexual positioning |  |  |  |  |
| *Insertive* | 2 | - | - | - |
| *Receptive* | 2 | - | - | - |
| *Both* | 2 | - | - | - |
| Ever used drug | 11 | 0.697 | 79.21 | <0.001** |
| Participated in group sex in the last 12 months | 2 | - | ^-^ | ^-^ |
| Condom Usage† |  |  |  |  |
| *With any male partners (Last Act)* | 12 | 0.337 | 88.67 | <0.001** |
| *With any male partners (Past 6 months)* | 8 | 0.621 | 88.29 | <0.001** |
| *With regular male partners* | 1 | - | ^-^ | ^-^ |
| *With casual male partners* | 1 | - | ^-^ | ^-^ |
| *With male clients (Last Act)* | 11 | 0.312 | 97.58 | <0.001** |
| *With male clients (Past 6 months)* | 9 | 0.677 | 94.83 | <0.001** |
| *With any female partners (Last Act)* | 3 | 0.602 | 82.6 | <0.001** |
| *With any female partners (Past 6 months)* | 3 | 0.117 | 18.6 | 0.293 |
| *With regular female partners (Past 6 months)* | 1 | - | - | - |
| *With casual female partners (Past 6 months)* | 1 | - | - | - |
| *With commercial female clients (Last Act)* | 1 | - | - | - |
| *With commercial female clients (Past 6 months)* | 1 | - | - | - |
| **III. HIV/STIs Testing Rates** |  |  |  |  |
| Ever tested for HIV | 8 | 0.026* | 95.27 | <0.001** |
| Tested for HIV in the past 12 months | 9 | 0.532 | 91.13 | <0.001** |
| Ever tested for STIs | 2 | - | - | - |
| **IV. Diseases Prevalence** |  |  |  |  |
| HIV | 16 | 0.322 | 66.67 | <0.001** |
| Syphilis | 15 | 0.198 | 66.29 | <0.001** |
| HIV-syphilis co-infection | 3 | 0.117 | 0.00 | 0.412 |

Note: Tests of publication bias and heterogeneity were not appropriate for subgroup meta-analyses with less than 3 studies. **p* < 0.05; ** *p* < 0.001.
