# Supplementary material for: HIV and Sexually Transmissible Infections among Money Boys in China: A Data Synthesis and Meta-Analysis
Source: PLoS One. 2012 Nov 29;7(11):e48025. doi: 10.1371/journal.pone.0048025 (PMC3510224; doi:10.1371/journal.pone.0048025)
Supplement: Table S4 — Result of individual variable meta-regression models for each subgroup meta-analysis. (DOCX) [file pone.0048025.s004.docx]

**Table S4: Result of individual variable meta-regression models for each subgroup meta-analysis.**

|  | **Study factors** | | | | |
| --- | --- | --- | --- | --- | --- |
| **Study Characteristics^f^** | **Sample size**  (≥100 *versus* <100) | **Language**  (Chinese *versus* English) | **Study Design**  (Non-venue-based *versus* venues-based) | **Study Method**  (Non-snowball sampling *versus* snowball sampling) | **Study Year**  (early 2000s *versus* late 2000s) |
| **I. Demographic characteristics** |  |  |  |  |  |
| Migrant | (dropped) | **b = -1.694**  ***p* < 0.001** | (dropped) | **b = -0.516**  ***p* < 0.001** | (dropped) |
| Current married | **b = -1.129**  ***p* = 0.095** | b = -0.198  *p* = 0.720 | (dropped) | b = -0.096  *p* = 0.837 | b = -0.183  *p* = 0.123 |
| Senior high school or above | b = 0.103  P = 0.462 | b = -0.0112  *p* = 0.930 | b = 0.352  *p* = 0.218 | b = -0.176  *p* = 0.070 | b = -0.057  *p* = 0.349 |
| **II. HIV-Related behaviours** |  |  |  |  |  |
| Sexual orientation |  |  |  |  |  |
| *Bisexual* | (dropped) | b = 0.315  *p* = 0.707 | (dropped) | b = -0.177  *p* = 0.793 | b = -0.125  *p* = 0.351 |
| *Homosexual* | b = 0.160  *p* = 0.700 | b = -0.185  *p* = 0.573 | b = 0.067  *p* =0.884 | b = 0.180  *p* = 0.452 | b = 0.028  *p* = 0.768 |
| *Heterosexual* | b = 0.010  *p* = 0.997 | b = -0.610  *p* = 0.755 | b = 0.138  *p* = 0.631 | **b = 0.745**  ***p* = 0.066** | b = -0.055  *p* = 0.874 |
| *Others* | b = 2.160  *p* = 0.102 | b = 0.018  *p* = 0.976 | (dropped) | b = -0.786  *p* = 0.253 | b = 0.086  *p* = 0.522 |
| Ever used drug | b = 0.291  *p* = 0.159 | b = -0.153  *p* = 0.536 | (dropped) | b = -0.184  *p* = 0.494 | b = -0.003  *p* = 0.974 |
| Condom Usage |  |  |  |  |  |
| *With any male partners(LA)* | b = 0.159  *p* = 0.375 | (dropped) | b = 0.355  *p* = 0.260 | b = -0.045  *p* = 0.800 | b = 0.073  *p* = 0.298 |
| *With any male partners(P6M)* | b = -0.032  *p* = 0.917 | b = 0.665  *p* = 0.137 | (dropped) | b = 0.227  *p* = 0.475 | (dropped) |
| *With male clients (LA)* | **b = 1.061**  ***p* < 0.001** | (dropped) | (dropped) | **b = -0.982**  ***p* < 0.001** | **b = -0.234**  ***p* < 0.001** |
| *With male clients(P6M)* | **b = -0.545**  ***p* < 0.001** | **b = 0.509**  ***p* < 0.001** | (dropped) | **b = 0.687**  ***p* < 0.001** | b = 0.0337  *p* = 0.150 |
| **III. HIV/STIs Testing Rates** |  |  |  |  |  |
| Tested for HIV in the past 12 months | b = -0.071  *p* = 0.911 | b = -0.038  *p* = 0.952 | (dropped) | b = -0.831  *p* = 0.258 | b = 0.023  *p* = 0.859 |

Note: ^f^ Only characteristics with high and significant heterogeneities (*I^2^ >* 75.00*, p <* 0.10*)* were analysed in the meta-regression model. Table showing the meta-regression coefficient (b) and significance of b (*p*-value). *p*-values in bold print represent significant associations (*p* < 0.10). Factors that were collinear with other study factors were dropped in the model.
